# Exploring the Experiences of Co-morbid Pain and Depression in Older African American Women and Their Preferred Management Strategies

**DOI:** 10.3389/fpain.2022.845513

**Published:** 2022-02-28

**Authors:** Brittany F. Drazich, Emerald Jenkins, Manka Nkimbeng, Martha Abshire Saylor, Sarah L. Szanton, Rebecca Wright, Mary Catherine Beach, Janiece L. Taylor

**Affiliations:** ^1^School of Nursing, University of Maryland, Baltimore, MD, United States; ^2^School of Nursing, Johns Hopkins University, Baltimore, MD, United States; ^3^School of Public Health, University of Minnesota, Minneapolis, MN, United States; ^4^School of Medicine, Johns Hopkins University, Baltimore, MD, United States

**Keywords:** pain, depression, African American, older adult, women

## Abstract

The intersection of race, gender, and age places older African American women at an increased risk for untreated physical pain and depression that can significantly diminish their quality of life. The objectives of this study were to (1) explore older African American women's perceptions of pain and depressive symptoms and how these symptoms influence each other, and (2) explore effective pain and depression alleviation strategies used by the women. We conducted five focus groups with older African American women (*N* = 18). We used deductive coding to analyze focus group transcripts and qualitative description to summarize themes. We identified five major themes: (1) Spiritual Suffering from Linked Pain and Depression, (2) Lack of Understanding from Healthcare Providers, (3) Push Through and Live Through, (4) Medications Not Worth the Risk and, (5) Strategies for Pain and Depression. This study offers insight into the experiences of pain and depression in older African American women, and alleviation strategies they perceive as effective. These qualitative findings may be used to inform interventions for older African American women who experience pain and depressive symptoms.

## Introduction

Women are significantly more likely to report pain and/or depression compared to men (1). These conditions frequently co-occur and can exacerbate one another (1, 2). The biopsychosocial model suggests that the cyclical relationship between pain and depression occurs due to interconnected psychological, biological, and social factors of health (3). For example, the pain experience adversely affects thoughts and behaviors (4). Women who are experiencing pain may suffer more negative thoughts or isolate themselves from others, contributing to depressive symptoms (1, 5, 6). Additionally, shared biological etiologies may explain the co-occurrence of pain and depression in women (5–7). For instance, levels of hormones such as norepinephrine and estrogen could play a role in pain perception, as well as depression (1, 7, 8).

Beyond biology, the intersection of the lived experience of African American race, female gender, and older age places older African American women at an increased risk for experiencing comorbid pain and depressive symptoms (9–14). Older African Americans experience higher rates of pain than non-Hispanic white older adults, with 55.6% reporting pain, and they are also prescribed pain medications less (11, 12, 15). Depressive symptoms among African Americans are more severe than other racial/ethnic groups, but only 45% of African Americans with Major Depressive Disorders receive treatment (16). Additionally, older African Americans are more likely to have undiagnosed and untreated depression compared to younger African Americans (17–19). This age disparity is largely due to the unique manifestation of depression in older adults (e.g., somatic), and some reticence among older adults to discuss mental health issues compared to physical health issues (17, 18). Although older African American women are more likely to experience pain and/or depression, there is a dearth of qualitative research exploring their perceptions of these co-morbid conditions.

In many cases, comorbid pain and depressive symptoms in older African American women contribute to the misdiagnoses, and under-treatment of either or both conditions (14, 20–22). Understanding the experiences of pain and depression among older African American women, as well as their perceptions and use of pain and depression interventions, can inform the development and tailoring of interventions for these conditions. Previous research suggests that older African Americans often prefer non-opioid analgesics and non-pharmacological interventions such as prayer/meditation (23–26). However, more research is needed to identify culturally appropriate strategies specifically preferred by older African American women (6). Given their societal roles, family expectations, and high rates of comorbid pain and depression, it is important that we identify strategy preferences specific to this population (27). To fulfill these research gaps, we conducted focus groups. These focus groups aimed to (1) explore older African American women's perceptions of pain and depressive symptoms and how these symptoms influence each other, and (2) explore effective pain and depression management/treatment strategies used by the women.

## Methods

After obtaining approval from the Johns Hopkins School of Medicine Internal Review Board we conducted a qualitative descriptive study.

### Participants

Women were eligible to participate if they (1) identified as African American, (2) lived in Baltimore, (3) were aged 50 years and older, (4) had a depression score of at least 5 on the Patient Health Questionnaire-9 (PHQ9) (28) at time of screening which indicates mild depression, (5) experienced pain self-rated as 3 or greater (0–10 scale), and (6) had pain that prevented them from doing at least one activity that they preferred to do. We chose 3 as the pain cutoff to capture anyone with “mild” pain on average; however, we also required that pain must be limiting at least one activity, indicating that the pain may at times increase in intensity. We conducted purposive convenience sampling through distributing flyers within organizations serving older African American women in Baltimore such as senior apartment buildings, churches, and beauty salons. We recruited 18 women who participated in one of five focus groups (3–4 women per focus group).

### Measures

We administered the PHQ-9, which is a validated and self-administered survey which scores 9 depressive symptoms from 0 “not at all” to 3 “nearly every day” (28). The PHQ-9 ranges from 0 to 27, with higher scores indicating greater depressive symptoms. We also administered three instruments from the Patient Reported Outcomes Measurement Information System (PROMIS) to obtain information on the women's pain: the Item Bank Pain Intensity Short Form (29), the Pain Interference Short Form, and the Pain Behavior Short Form. These surveys measure pain intensity (scored 3–15), pain interference (scored 8–40), and pain behavior (scored 7–35), respectively, with higher scores indicating more pain symptoms (28–30). These PROMIS instruments have been validated in large samples (30).

### Procedures

At the start of each focus group, we re-administered the PHQ-9 (28) and administered the three PROMIS surveys described above to obtain information on current pain and depressive symptoms. Thus, we administered a different pain measure at study screening and at the start of each focus group. The purpose of the administration of these instruments at the start of each focus group was to describe the sample.

We conducted focus groups rather than individual interviews to spur interactions and discussion among the participants regarding their common conditions of pain and depressive symptoms (31). Four of the focus groups were held at a church central to the East Baltimore community and the fifth (final) focus group was held at a Johns Hopkins health building within the East Baltimore community. The moderators (Authors 1–3) used a focus group guide developed by the senior author. The moderators were all female, had no prior relationship with the study participants, and had no personal investment in the study results. The focus group guide focused on participant experiences/beliefs about pain and depressive symptoms and strategies or techniques to address pain and depressive symptoms (4). The guide also explored the unmet needs of the participants surrounding pain, depression, and communication with healthcare providers about these health needs. Example questions included, “Do your depressive symptoms keep you from doing any activities?” and “Do you think there are strategies that can be helpful for both pain and depressive symptoms or are they separate?” The basis for these questions stemmed from our previous work (4, 9, 12). After each focus group, the study team met to record an audit trail, discuss the appropriateness of the guide, and take notes on any changes that needed to be made. Following the first focus group, we edited the focus group guide for improved clarity but did not add any questions. Focus groups lasted approximately 75–90 min, were audio recorded, and transcribed verbatim by a professional service. During the focus groups, participants referred to each other by their designated participant number to maintain anonymity for the recording, and any identifying study materials were stored in a locked cabinet. No participants left or dropped out during the focus groups. In appreciation for their time investment during the focus groups, participants were compensated $20.

### Analysis

We used deductive coding to analyze focus group transcripts and qualitative description to summarize themes. Qualitative description emphasizes “staying close to the data,” which enabled us to focus on straight descriptions of the experience of pain among African American women (32, 33). Authors 1–3 and the senior author met to create the deductive codebook, constructed to reflect the interview guide, study objectives, and field notes. Two authors (Authors 1, 3) independently applied the codes to the five focus group transcripts, adding additional codes that emerged from the data. All data entry and coding occurred using the qualitative coding software f4analyse (34). Saturation, defined as redundancy of data with no newly emerging themes, was achieved after the third focus group (35). Two additional focus groups were conducted to confirm saturation. After coding all five transcripts, Authors 1 and 3 compared their codes using a side-by-side display to establish consensus, and created a coding tree to synthesize codes into categories and categories into themes (36). Authors 1 and 3 then met to discuss rival explanations, or alternative perspectives to interpreting the data, and collectively agreed upon the results (37, 38). Considering the post-positivist view held by the research team, reflexive dialogue was conducted to identify the judgements, practices, or biases of the researchers that might affect study conclusions (39, 40).

## Results

The average pain intensity rating at the time of the focus group was 9.5 out of a possible 15, and the average PHQ-9 score at the time of the focus group was 8.4 out of a possible 27, indicating mild depressive symptoms (Table 1). The average age of the women was 61.9 years, although four participants stated they were 50 or over but declined to give their ages. Analysis of focus group transcripts identified five major themes: (1) spiritual suffering from linked pain and depression, (2) lack of understanding from healthcare providers, (3) push through and live through, (4) medications not worth the risk, and (5) non-pharmacological strategies to manage pain and depression. Table 2 includes a list of quotes and excerpts showing support of the respective themes. The focus group findings are presented below.

**Table 1:** Participant demographics.

	***n***	**Mean (SD)**
Age	14	61.9 years (5.1)
50–55	2	
55–60	3	
60–65	6	
65–70	3	
PHQ-9	18	8.4 (5.3)
Pain intensity short form 3a	18	9.5 (3.0)
Pain interference short form 8a	18	23.1 (7.8)
Pain behavior short form 7a	18	27.5 (4.4)

**Table 2:** Representative excerpts from the focus groups.

**Theme**	**Pseudonym**	**Representative quote**
Spiritual suffering from comorbid pain and depression	*P18*	“*When you don't have casts and stuff that people can see they don't understand what's wrong with you, because they don't see something wrong with you, and so I just stayed to myself, because I got tired of explaining the horrible pain that I was in, and so the pain was bad, but then the deep depression that I got into made it even worse. I didn't want to live anymore. I did not want to live, because I didn't know how to navigate the way my body was feeling, so, yeah, I attempted suicide.”*
	*P4*	“*And then when you have a lot of pain it's like you're not living. You don't have a life. All you got is this pain to look forward to every day when you wake up. That is not fun. You know?”*
	*P5*	“*… the pain can make you like you want to but you can't and that's depressing right there. You want to. I like dancing. I like doing different things. I can't get out there if my hip is hurting, you know, if my hip is hurting and stuff. That's depressing. I can't live my life, I think, to its fullest.”*
Lack of understanding from healthcare providers	*P7*	“*Some doctors you're really afraid to even talk about things... Or they really don't care that much. They're not really interested in because you got a lot of pain.”*
	*P19*	“*I have another doctor now, and the communication is good listening to her and everything, but I have a feeling that she doesn't really understand everything that I ‘m saying in the way that I want her to understand.”*
	*P18*	*I told him “I have been in therapy since ‘91. I know the difference. This is real.” So I just grabbed up my papers, walked right out of there, went to the information desk and said “I need someone who knows that fibromyalgia is real,” okay, so that was the only time I had an adverse– that there was no communication. He just said “I don't believe in fibromyalgia. Okay?”*
Medications not worth the risk	*P19*	“*I have a history that I have to consider before I take medications, so I try to avoid narcotics, because I never want to go back there again… “I had a doctor one time try to put me on a medication that was definitely habit-forming and could do even more damage in the long run, so I got some help to get off the medication she had put me on, and I now don't allow any doctor to give me anything like that.”*
	*P10*	“*A doctor suggested Percocet. I told that doctor to keep that prescription. The OxyContin's and all of that those doctors don't write it because they know I'm not going to take it.”*
	*P16*	“*I was taking amitriptyline and gabapentin and then Abilify, and it was making me depressed.”*
	*P6*	“*And I think it's an honor and a pleasure that somebody cares enough to say, “Well, let's find out what we can do to try to help some type of way without medication.” It's always medications. But it's another way with the mind. That little mind up there is a powerful tool.”*
Push through and live through	*P4*	“*Sometimes you just go to push through it. And I'm not saying push through every situation. But some stuff that got to get done, push through that pain and keep it going because that pain is going to be there no matter what. You know? That's my feelings on that.”*
	*P11*	“*But I try to push myself to do a little work out or exercises because if I stand still or lay still then it's going to stiffen up really bad on me.”*
	*P9*	“*So I like put it on the back of my mind that I don't have this pain. So it's not bothering me right now. That take it back to a mind over matter.”*
	*P16*	“*Please don't get it twisted. It hurts, but I just can't sit home. You need to move anyway.”*
Non-pharmacological strategies for pain	*P13*	“*Let go—that serenity prayer. Give it to the master, the higher being. I can't carry it. So it's a new day. So I really don't have any problem in managing my pain.”*
and depression	*P4*	“*…if you go to the pool and there are other people that kind lifts your spirit up, too, because sometimes they're going through the same things that you're going through.”*
	*P5*	“*But sometimes it's so depressing when you can't do things and you're by yourself. And I'll go to these groups. And it's like we talk, just different subjects. We may do an activity or whatever. Just the idea sometimes being in that group with other people that's not going through what I'm going through but everybody has got something that's going on with them.”*
	*P9*	“*I find a lot if you go to sleep with pain or you feel that pain drink yourself a cup of tea, nice hot bath or shower; I'm going to say bath, something you can sit in that water and feel it. With a little extra soap, nice hot cup of tea or whatever, relax that mind and that body before you climb into the bed, you'll feel 100 percent better.”*

### Spiritual Suffering From Linked Pain and Depression

In this study, the majority of the women identified a link between their pain and depression, most often with pain as a precursor for depression. They described the ways in which pain sometimes directly contributed to their depression. For example, the women offered stories of pain so unrelenting, burdensome, and severe that they experienced depression, and in two cases, suicidal ideation. In other cases, the women believed pain indirectly contributed to their depression. For instance, they attributed pain to both sleepless nights and self-isolation, which in turn affected their mood. Pain also indirectly contributed to their depression through life interference. The women discussed the sadness that they felt ruminating on their limitations resulting from pain. They found it frustrating that their pain was not visible, and therefore, obvious to others. One woman expressed how she felt unable to engage in aspects of life that used to bring her joy:

“When I was first diagnosed with fibromyalgia in 1995, the onset was so severe that I couldn't do anything…I didn't understand my body because I was used to being active, jogging, running, playing tennis, and I couldn't do any of those things anymore, and that turned to major depression because I couldn't relate to the world anymore.” (P18)

The women described that this cycle of pain and suffering had implications for their ability to see meaning and experience holistic fulfillment. As they experienced physical limitations from their pain, they often became emotionally and mentally distressed/depressed, which at times led to additional physical pain. This cycle went beyond pure mental and physical suffering and was explained in terms of how it influenced the “spirit” by several participants. For example, participant 6 stated:

“When you have pain you get depressed… when something is a discomfort it brings you in a down spirit mode.”

Additionally, participant 12 stated:

“Now for me, the pain that works with depression for me is spiritual. It's spiritual pain. It's not physical pain. ”

### Lack of Understanding From Healthcare Providers

Many of the women expressed that their healthcare providers did not understand the severity of their pain and depression. Although the majority of the women reported that they had developed respectful and positive relationships with their providers, they nonetheless felt these providers lacked an understanding of the gravity and impact of their comorbid symptoms. For example, participant 19 stated:

“I have another doctor now, and the communication is good listening to her and everything, but I have a feeling that she doesn't really understand everything that I'm saying in the way that I want her to understand.”

The participants expressed that this lack of understanding had implications on their providers' decision-making surrounding treatment choices and follow-up care, inhibiting their healing. Their providers would often wait for severe clinical symptoms of depression to manifest before taking action. In one case, it took a near fatality for a provider to recognize the gravity and distressing impact of pain as participant 5 explained:

“My primary doctor didn't really know how depressed I was about everything, because I've been through a couple of cancers and surgeries, until I tried to commit suicide.”

The participants also conveyed their vulnerability when broaching the topic of pain with their health provider, explaining that they sometimes felt “afraid” that their pain experience might not be well perceived. Some participants expressed worry that the health provider might judge them for their health history, rather than caring about their current well-being. For example, participant 4 conveyed a feeling of being judged by their provider:

“And sometimes people don't really understand your pain. Only you can truly understand it. And your healthcare provider sometimes thinks that you're whining.”

### Push Through and Live Through

The women expressed that pain would be present on most days; therefore, they should push themselves to complete the social roles expected of them. Although they might not be able to ignore the pain, they could “still” or quiet their pain by compartmentalizing it into the back of their minds. They could push through the pain using willpower and “mind over matter.” For example, participant 8 relayed:

“If you know your body hurts [but] it's something that you really, really want to do. I like to psych myself out. I just keep going. ”

They also pushed themselves to compartmentalize symptoms associated with depression. Often the initial push was the catalyst needed to maintain momentum for the rest of the day. They noted that if they did not push themselves, they risked lying in bed all day, which exacerbated pain, stiffness, and depressed mood. The women identified that they had to live through it regardless of the debilitating nature of their comorbid pain and depression. Although the women experienced this cycle of pain and depression, they had to oftentimes push themselves both mentally and physically each day to function, otherwise they would disengage completely.

### Medications Not Worth the Risk

The women discussed their past utilization, and concerns with, pharmacological pain and depression alleviation techniques. With the exception of two focus group participants, the majority of the women were distrustful of narcotics. Every focus group had participants who expressed a desire to avoid addiction when discussing pain medication. Many women had personal experiences with addiction, expressing their belief and fear that they would experience addiction again if they were not careful about avoiding narcotics. Participant 12 said:

“If I take a pill and, whew, it take away all this pain, then I'm going to be popping them pills all the time. So I'm afraid to do that…”

The women also disliked the side effects of various types of pain medication and depression medications such as feeling dizzy, high, or experiencing swelling. For example, participant 18 stated:

“I don't take anything for pain, because I don't like feeling doped-up.”

Due to the potential for addiction and pain medication side effects, the women preferred to use over-the-counter medications or creams, non-pharmacologic pain alleviation techniques, or simply “deal with the pain.” They expressed differing views on the effectiveness of over-the-counter medications. The women were generally open to working with a pain specialist in the future, provided the pain specialist focused on non-pharmacological pain alleviation techniques. Although participants seemed more trusting of antidepressant medications than prescription pain medications, some participants similarly conveyed their concern regarding antidepressant medication side effects such as a decreased awareness of their surroundings.

### Non-pharmacological Strategies Utilized for Pain

The women discussed their past utilization of non-pharmacological pain alleviation techniques, which were generally effective. They spoke positively of exercise-based strategies to improve pain such as yoga, stretching, and Zumba, and had especially high opinions of water therapy. One woman referred to her water aerobics class:

“So that time in that water really does help your body. And when you don't go, missing days, you feel it. You feel it. Yes, I love it.” (P5)

They admitted that exercise might hurt initially but over time loosened their stiff muscles. Exercise also contributed to weight loss, which can improve pain on their back and knees. The women also described methods they used to distract their mind and subsequently improve their pain. For example, meditation, coloring, watching comedy, or listening to nature sounds/music, helped them clear their minds from their pain. One woman even expressed that her meditation relieved the pain during her heart attack. The women also experienced pain alleviation from physical techniques such as massage and heat therapy (drinking or holding hot tea or taking long baths). They stated that cold therapy could also be helpful, but the relief experienced was fleeting. Lastly, the women expressed that the knowledge gained and activities performed during support groups was also an effective pain alleviation technique.

### Non-pharmacological Strategies Utilized for Depression

The women described depression alleviation strategies that they found effective in improving their depressive symptoms such as changing their behavior, support groups, and spirituality. They changed their behavior through both avoiding situations that lowered their mood, and forcing themselves into activity. For example, the women recounted how working out at the gym, going for a walk, or meditating improves their depression. They believed that maintaining an occupied mind and body was key to a positive outlook. One woman said:

“Anyway, I find that once I get out and about everything changes. Your outlook becomes different. You're out, you're doing. That mindset changes totally so you're more inclined to do things.” (P17)

Similarly, one woman explained how adopting a dog forced her to get active by providing care, in turn improving her depression. The women also identified community-based assistance for overcoming depression such as being around others, receiving counseling, and support groups. The women described how the act of being in the company of other people and sharing experiences improved their mood. Support groups and community were commonly discussed outlets that provided help for the women. One woman explained that often friends or family are uncomfortable discussing depression due to stigmatization. Alternatively, the women perceived that support groups can provide a safe place to discuss depression and share similar experiences, often improving their mood. Participant 14 stated:

“I remember coming together having groups like this, because it doesn't exist anymore, that I know of. And they're needed. Especially with depression, because depression is large, and so many of us don't admit to it.”

Lastly, the women believed that spirituality was an effective depression alleviation strategy, finding strength from God or their church community. Of note, many of the depression alleviation strategies discussed by the women in the focus groups were also mentioned as useful pain alleviation strategies (e.g., support groups, spirituality, and meditation).

## Discussion

The objectives of this study were to (1) explore older African American women's perception of their pain and depressive symptoms and how they believe they influence each other, and (2) determine what pain and depression strategies the women found effective. The major themes that emerged from the focus groups were: (1) spiritual suffering from linked pain and depression, (2) lack of understanding from healthcare providers, (3) push through and live through, (4) medications not worth the risk, and (5) non-pharmacological strategies to manage pain and depression.

The findings align with the biopsychosocial framework (3). For example, the women described how the pain symptoms (biological), contributed to a withdrawal from activities (social), which affected their mood (psychological and perhaps biological). Similarly, the women described that strategies to alleviate pain symptoms (biological) could be encouraged through supports from outside sources (psychological) or attending exercise and support groups (social). The connections between biological, psychological, and social factors of health (3) were evident through the focus groups, can help explain the link between pain and depression (1, 5–7), and can offer insight into solutions for dealing with these co-morbidities. Interestingly, the results indicated that the directionality of the link between pain and depression was often consistent. The women were more apt to describe pain leading to depression, rather than depression leading to pain. Complementing this finding, previous research indicates that pain may adversely affect the quality of life of African American older adults more than White older adults, perhaps due to disparities in treatment access and the quality of medical care that they receive (41).

The findings from this study complement current literature on linked suffering of pain and depression (42, 43) and strengthen our understanding of the needs of older African American women who experience these conditions. The women described the blurring of boundaries between pain and depression, and a “spiritual suffering” from a combination of both conditions. According to Elison, the spirit of a person is the entity that equips people to see meaning and identify or require morality or equity (44). The women in this study expressed that their pain and depression influenced their spiritual health, which may further influence how they perceive, respond, and engage with others and their environment. The spiritual suffering they described goes beyond daily discomfort, influencing all aspects of who they are as African American women, and their overall well-being (44). Previous research suggests that when African American women, especially those who are older, have a sense of spiritual strength, it can help them psychologically cope during times of stress (45). With this spiritual strength compromised through comorbid pain and depression, their relationships, roles, and spiritual identities were impacted as well.

Based on the women's experiences with health care providers, the findings demonstrated that at times health care providers dismissed signs of pain and depression or failed to recognize the severity of symptoms. When patients feel dismissed by clinicians, they may become distrusting or disengaged with care (46). This has implications for mental health care utilization among African American women. Perhaps this care gap they experience contributes to the underdiagnosis and undertreatment of depression among community-dwelling older adults (17–19). As reflected in the focus groups, pain can contribute to depressive symptoms, and in some circumstances, even suicide (47). Given these associations, it is essential that community-based providers screen for depression in chronic pain patients and pain in patients with depressive symptoms. Additionally, clinicians should provide an accepting environment where patients feel willing to share their concerns without being judged.

In this study, the women described how they developed an ability to push through and live through their pain. This “pushing through” was a coping and survival mechanism in which they drew on personal resiliency reserves to fulfill their social roles. Previous research by Booker and colleagues identified a similar finding in that “dealin' with it” or “bearing the pain” was a common coping mechanism among African Americans with osteoarthritis (25). African American women pushing their bodies could have biological and psychological implications that contribute to suffering, such as further injury (48). Pushing through within the context of culture may be related to “Strong Black Woman” expectations within American society (49). Within this societal expectation, it is believed that African American women should be strong, resilient, and self–sacrificing (49, 50). This expectation on African American women may increase their risk for further experiencing depressive symptoms (50). An alternate or “rival” explanation for the “push through” theme is that the participants' concern with pharmacological addiction and side effects led them to believe that treatment was not an option, and thus, they must “push through” their pain and depressive symptoms to experience life. Additional work is needed to understand the cultural components, impacts, and outcomes related to this type of response among older African American women with comorbid pain and depression.

As mentioned above, focus group findings shed light on preferred forms of pain and depressive symptoms alleviation strategies among older African American women. Consistent with another study, the women identified that the risks outweighed the benefits of pharmacological pain management (51). As a whole, the women conveyed a preference for non-pharmacological pain alleviation strategies. Study participants who had experienced previous addiction were especially vocal of their distaste for narcotic pain medication. This study highlights the importance of health providers having a diverse array of treatment plans for their patients with pain, with deliberate inclusion of over-the-counter medications and non-pharmacological pain treatment options. In our previous work, we identified that older women with disabilities expressed not having a clear understanding of side effects of prescribed medications (4). It is important that older African American women have clear communication and information regarding medications for pain and depression in order to make the best informed decisions regarding their treatments. In addition, identifying the risk profile for certain medications may be necessary in order to tailor their care and best address their individual needs. Considering that older adults are at high risk for side effects from pharmacological interventions and often do not experience full relief from them (52), the participants' negative perceptions of pain and depression medications underlines the importance of developing and testing non-pharmacological treatments for these conditions among older adults. Although previous research indicates that older African American women utilize counseling services less than adults younger than 50 years (53), our sample of women verbalized an openness to support groups, especially those with a faith-based component.

### Research and Clinical Implications

These focus groups indicate the importance of addressing both pain and depression in older African American women through intervention and can guide such interventions (53). This study suggests that interventionists might consider the cyclical relationship between pain and depression, and approaches to breaking this cycle. For example, with the cycle of pain contributing to depressive symptoms because of limitations imposed by the pain (54), interventions that target these limitations/restrictions, such as exercise alternatives (e.g., water therapy) or adaptations, might contribute to the alleviation of both symptoms. Additionally, pain and depression interventions for older African American women should focus on communication with health care providers, harness existing coping mechanisms/resiliency of women such as compartmentalizing pain, while also supporting preferred non-pharmacological treatments such as leaning on community support or social connection. More specifically, this study can provide a “toolkit” of tangible depression and pain alleviation strategies that this sample of older African American women have previously perceived as effective. The participants could select strategies from this “toolkit” such as exercises, distraction, changing behavior, support groups, or activities that support their spirituality.

Limitations of this study include social desirability bias and sampling bias. During the focus groups, the women might have felt swayed by the opinions of the other group members, or by their expectations of what the moderator wanted them to say. We attempted to limit social desirability bias through establishing rapport and making clear our desire for honest responses (54). Another limitation is that some snowball sampling occurred in which focus group participants recruited their acquaintances to participate in the study. Snowball sampling can be a useful tool in recruiting difficult to reach populations, but can limit the diversity of perspective and can increase social desirability bias (55). Considering this sampling method, as well as the specific demographics of the study sample, this study's findings might have low transferability to other populations. Lastly, the women who participated in the focus groups verbalized depressive symptoms, but were not necessarily diagnosed with depression by a provider. Women without a formal diagnosis of depression may have different experiences than women diagnosed with depression by a clinician. Despite the limitations this study significantly contributed to the knowledge base on current perceptions, alleviation strategies, and unmet needs of older African American women with comorbid pain and depression.

## Conclusion

The intersection of race, gender, and age places older African American women at an increased risk for undertreated depression and pain that can significantly diminish their quality of life (10–12). The scarcity of literature and interventions related to culturally appropriate, non-pharmacological strategies to manage depressive symptoms and pain for older African American women may serve to perpetuate disparities in access to treatment and in the health and well-being of this group (6). This study fills this gap in research, and offers insight into the link between pain and depression in older African American women, and strategies they perceive as effective.

## Data Availability Statement

The de-identified data supporting the conclusions of this article will be made available by the authors, without undue reservation.

## Ethics Statement

The studies involving human participants were reviewed and approved by Johns Hopkins School of Medicine Internal Review Board. The patients/participants provided their written informed consent to participate in this study.

## Author Contributions

JT conceived the presented idea. JT and EJ conducted the focus groups. BD and MN performed qualitative data analysis. BD, MA, MB, and JT led the manuscript writing with support from all authors. All authors contributed to the article and approved the submitted version.

## Funding

This work was supported by the Johns Hopkins Building Interdisciplinary Research Careers in Women (BIRCWH) (NIH ORWH: 3K12HD085845-03S1) and the Johns Hopkins Older Americans Independence Center (NIA: P30AG021334). BD was supported by the Robert Wood Johnson Future of Nursing Post-doctoral Scholars Program. MN was supported by the Robert L. Kane Endowed Chair at the University of Minnesota School of Public Health. JT was supported by the Robert Wood Johnson Harold Amos Medical Faculty Program.

## Conflict of Interest

The authors declare that the research was conducted in the absence of any commercial or financial relationships that could be construed as a potential conflict of interest.

## Publisher's Note

All claims expressed in this article are solely those of the authors and do not necessarily represent those of their affiliated organizations, or those of the publisher, the editors and the reviewers. Any product that may be evaluated in this article, or claim that may be made by its manufacturer, is not guaranteed or endorsed by the publisher.
